# Hereditary leiomyomatosis and renal cell carcinoma: a case report and literature review

**DOI:** 10.3389/fruro.2026.1907141

**Published:** 2026-08-20

**Authors:** Shuqi Zuo, Yafei Xue, Fei Wang, Honglin Guo, Min Cui, Xingbo Zhao, Xiaoyi Qi

**Affiliations:** 1 Department of Obstetrics and Gynaecology, Shandong Provincial Hospital Affiliated to Shandong First Medical University, Jinan, Shandong, China; 2 Department of Pathology, Shandong Provincial Hospital Affiliated to Shandong First Medical University, Jinan, Shandong, China; 3 Department of Obstetrics and Gynaecology, Shandong Provincial Hospital, Shandong University, Jinan, Shandong, China

**Keywords:** FH gene, FH-deficient renal cell carcinoma, hereditary leiomyomatosis and renal cell carcinoma (HLRCC), metastasis, uterine leiomyoma

## Abstract

Hereditary leiomyomatosis and renal cell carcinoma (HLRCC), caused by germline *FH* mutations, is a rare autosomal dominant syndrome. This report details a 42-year-old woman with aggressive FH-deficient renal cell carcinoma (RCC) and multiple uterine leiomyomas. Radical nephrectomy and subsequent hysterectomy confirmed FH-deficient tumors via immunohistochemistry and genetic testing (*FH* c.1240A>G, p. Lys414Glu). Despite adjuvant immunotherapy and targeted therapy, rapid bone metastasis occurred postoperatively. This case highlights the aggressive nature of HLRCC-associated RCC, underscores challenges in therapeutic management, and emphasizes the necessity of early genetic testing and multidisciplinary surveillance to improve outcomes.

## 1 Introduction

HLRCC is a rare autosomal dominant disorder caused by germline pathogenic variants in the fumarate hydratase (FH) gene (1), detectable in 90% of affected families. Our systematic review of 92 English-language publications in PubMed reveals this syndrome manifests as a diagnostic triad (1): cutaneous leiomyomas (2), uterine smooth muscle tumors (present in 73-100% of female carriers) (2, 3), and (3) FH-deficient renal cell carcinoma (RCC) – now designated as a distinct tumor entity in the current WHO classification of urinary and male genital tumours. Kidney tumors in patients with HLRCC are typically unilateral and isolated, and HLRCC-associated renal cell carcinomas are the most aggressive types of kidney cancer, characterized by early metastasis (even if the primary tumor is small) and poor clinical prognosis (4, 5). We present a 42-year-old female carrying a pathogenic *FH* c.1240A>G variant resulting in p. Lys414Glu (K414E) substitution, demonstrating the classic phenotype: (1) multiple symptomatic uterine leiomyomas (menorrhagia, infertility), (2) stage pT3a renal tumor with papillary and tubular structures, and (3) metastatic progression within 12 months post-nephrectomy.

## 2 Case reports

**Figure 1:**
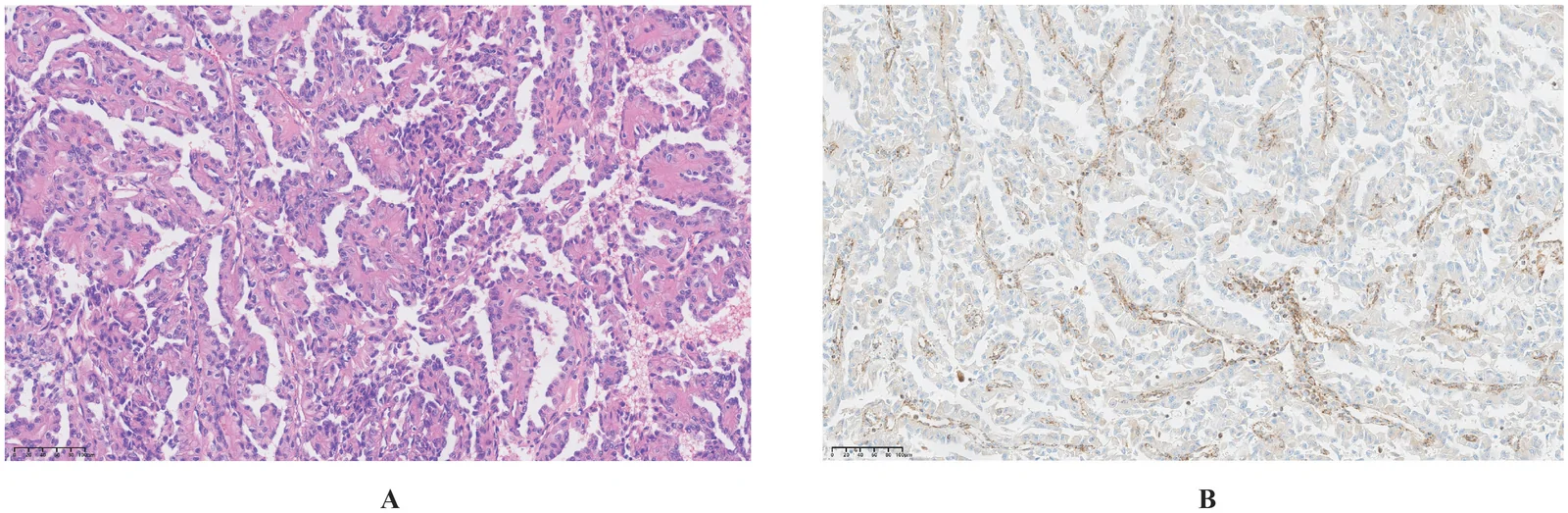
Pathological images from renal carcinoma specimen. **(A)** H&E staining: renal cell carcinoma with papillary and tubular structures (200x). **(B)** Fumarate hydratase immunostain: loss of expression in the tumor cells (blood vessel shows retained FH, internal control) (200x).

A 42-year-old woman presented with two-month progressive abdominal distension. Her surgical history included two myomectomies (2008, 2012) for uterine leiomyomatosis. The proband’s mother had a history of multiple uterine leiomyomas. Her children are currently young and have not manifested any symptoms associated with HLRCC to date. Information regarding other relatives was unavailable, as the proband declined to disclose or was unaware of their medical histories. In 2023, surveillance imaging detected a left renal mass, prompting urology referral. She denied hematuria, flank pain, or voiding dysfunction. Contrast-enhanced CT showed a 10 cm Bosniak IV left renal mass with tumor thrombus extending into the left renal and gonadal veins. Laparoscopic radical nephrectomy with thrombectomy confirmed FH-deficient RCC (pT3aNxMx; 10 cm; Fuhrman grade 2-3, renal pelvis/capsular invasion, venous thrombus with clear margins). Immunohistochemistry showed PAX8 (+), TTF1 (-), CD10 (-), Melan-A (+), CA9 (+), Ki-67+ (hotspot 20%), P504S+ (focal), TFE3 (-), FH (-), HMB45 (-), CK7 (-) (**Figures 1A, B**). Although CA9 positivity is often associated with clear cell RCC, it has been documented in FH-deficient RCC as well (6); the predominant papillary and tubular morphology in our case supported the diagnosis of FH-deficient RCC rather than clear cell RCC. The urologist recommended genetic testing, but the patient declined for personal reasons.

**Figure 2:**
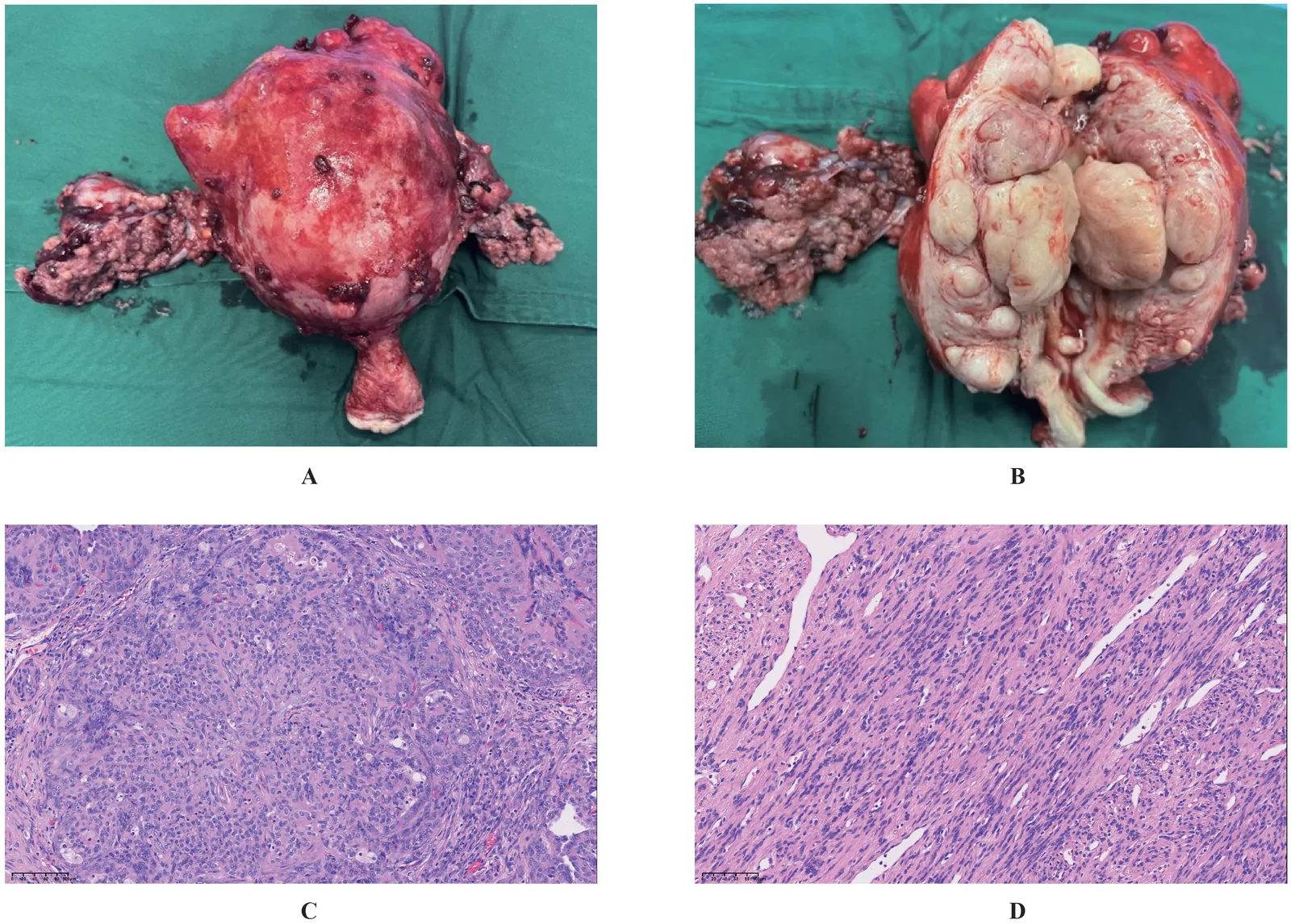
Intraoperative and pathological specimens. **(A)** Holistic view of uterus and bilateral adnexa. **(B)** Section view of uterus and bilateral adnexa with multiple uterine fibroids. **(C)** Ovarian cancer lesion (H&E, 200×): highly atypical papillary tumor cells resembling HLRCC-associated renal cancer. **(D)** Fibroid tissue (H&E, 200×): nucleo-oval tumor cells in chain pattern, staghorn vasculature; eosinophilic nucleoli with perinuclear halos.

**Figure 3:**
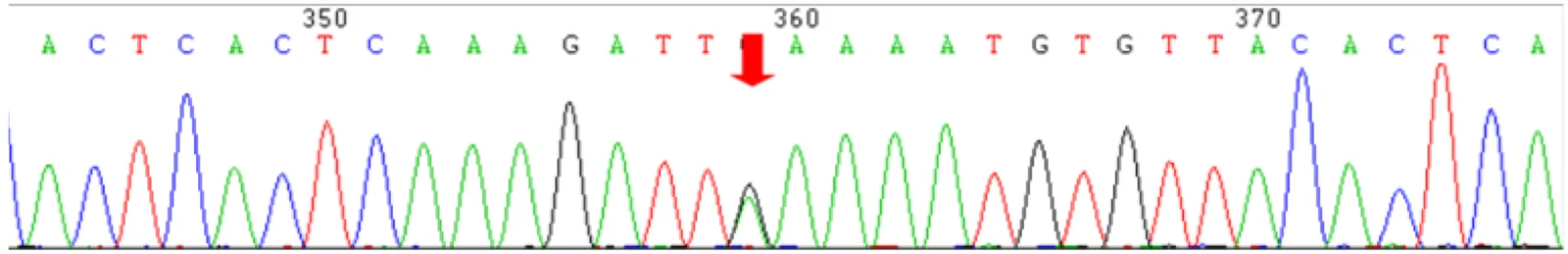
The genetic results of the switched germ line. FH NM-000143: c.1240A>G/p. (Lys414Glu).

July 2024 ultrasound revealed pelvic solid masses (hypoechoic ovarian/pelvic nodules) with lymphadenopathy, ascites, and multifocal uterine leiomyomas. Serum CA-125 was elevated (522 U/mL). Exploratory laparotomy with 4,000 mL ascites drainage showed a 5-month gestation-sized uterus with surface leiomyomas, bilateral ovarian masses, and peritoneal lesions (**Figures 2A, B**). This was followed by abdominal total hysterectomy, double adnexectomy, omentectomy, and pelvic metastatic resection. Pathology confirmed metastatic renal cell carcinoma (fallopian tube, parametria, pelvic floor, omentum) and FH-deficient leiomyomas (Desmin+/FH–) (**Figures 2C, D**). Immunohistochemical results: Uterine leiomyomas: Desmin (+), FH (-) (confirming HLRCC-associated pathology); Ovarian tumors: PAX8 (+), CA9 (+), RCC (+), WT-1 (-), ER (-), PR (-), CD10 (-), CK7 (-), CD117 (-), P504S (+), Ki-67+ (30% hotspot), FH- (partial). Postoperative germline genetic testing (performed on blood DNA) identified an FH variant (c.1240A>G/p. Lys414Glu) (**Figure 3**). Sequencing of the renal tumor or leiomyoma to assess for loss of the second FH allele was not performed. Intraperitoneal carboplatin (August 2024) reduced CA-125 to 99 U/mL (September 2024). Enrollment in a lenvatinib/tirelizumab trial was followed by bone metastases at 3 months, prompting denosumab therapy. At the time of publication of this article, the patient was continuing denosumab therapy (administered subcutaneously every 4 weeks) for bone metastatic lesions, alongside regular imaging surveillance and laboratory assessments performed approximately every 3 months to evaluate disease status and guide subsequent treatment decisions.

**Figure 4:**
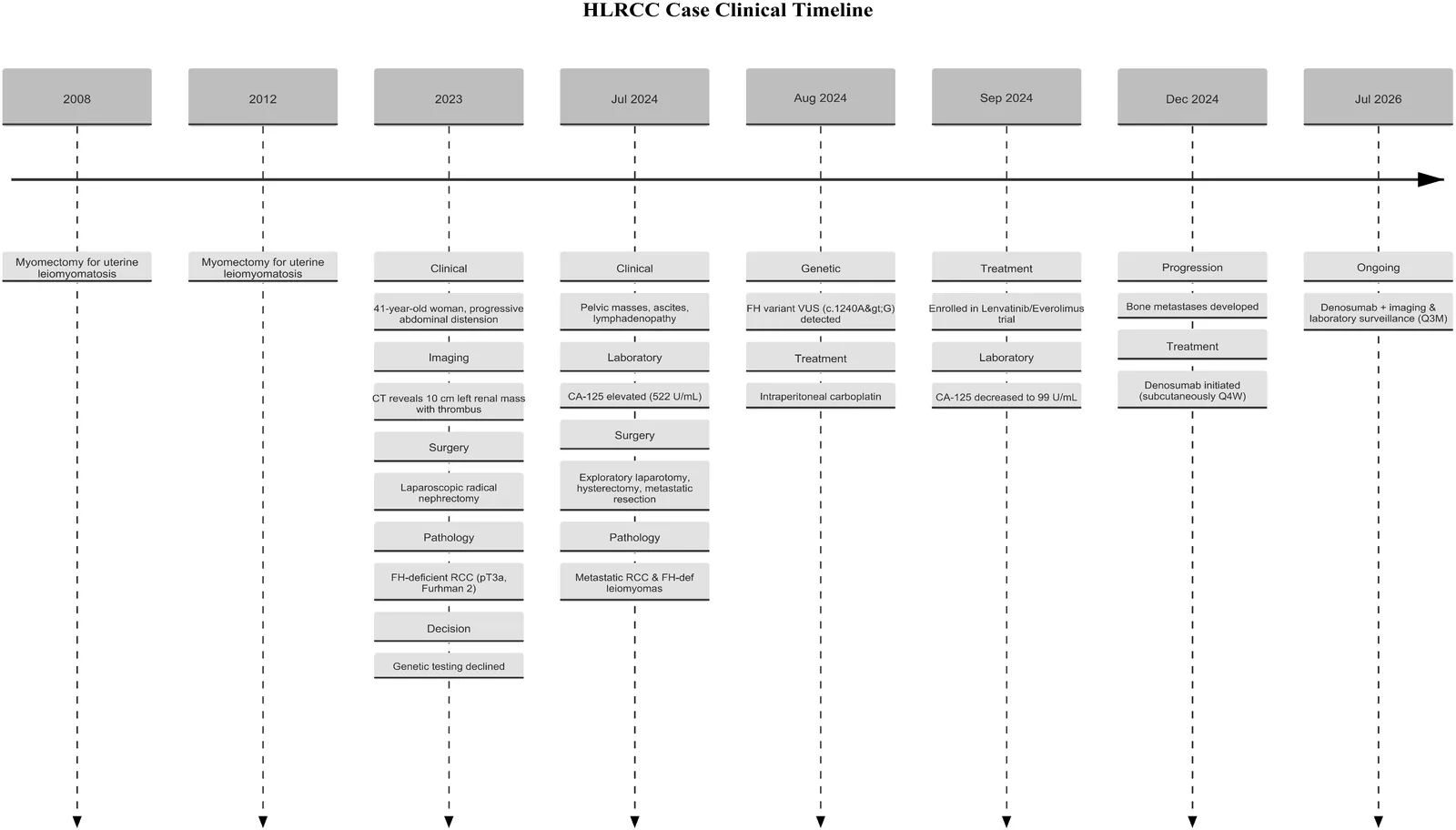
Clinical timeline. This chart shows the chronological sequence of clinical manifestations, imaging findings, surgical interventions, systemic treatments, and key laboratory results (CA-125) from first presentation to the most recent follow-up, illustrating the aggressive course of HLRCC-associated RCC.

The complete diagnostic and therapeutic course is presented chronologically (**Figure 4**).

## 3 Discussion

We describe a female HLRCC patient who underwent radical left nephrectomy for RCC, followed by hysterectomy and bilateral salpingo-oophorectomy one year later for pelvic peritoneal metastases and uterine fibroids. HLRCC typically presents with cutaneous leiomyomas (smooth pigmented papules/nodules) or early-onset uterine leiomyomas; these may occur alone or with RCC. While our case cutaneous lesions were absent here, FH deficiency on immunohistochemistry, characteristic uterine leiomyoma morphology, and renal imaging confirmed the HLRCC diagnosis. Among women with *FH* gene mutations, 73% to 100% develop uterine leiomyomas (2). Fumarase-deficient uterine leiomyomas account for only 0.4%–1.6% of all uterine leiomyomas (7). FH-deficient uterine leiomyomas are characterized by early onset, high recurrence risk, and distinct morphology: staghorn vasculature, alveolar-like stromal edema, prominent nucleoli with perinuclear halos, hypercellularity, nuclear atypia, and cytoplasmic hyaline globules—all observed in our case (8, 9). Renal cell carcinoma occurs in 20%–34% of HLRCC families (2). Some studies suggest that type 2 papillary renal cell carcinoma (PRCC2) may be the sole manifestation of HLRCC (10). Although historically described as type 2 papillary RCC, these tumors are now uniformly classified as FH-deficient RCC under the 2022 WHO system. HLRCC-associated renal lesions exhibit diverse radiographic presentations, but most are unilateral and isolated, demonstrating high T2 signal intensity, heterogeneity, and well-defined margins. However, contrary to the description of limited spread, these tumors are clinically aggressive; nodularity is present in approximately 50% of cases, and early metastasis is a well-recognized feature of HLRCC-associated RCC, underscoring the need for prompt surveillance and intervention (11). This case illustrates concurrent uterine leiomyoma and renal cell carcinoma in HLRCC.

Fumarate hydratase (FH) deficiency triggers pathological fumarate accumulation, fostering a protumorigenic microenvironment via three mechanisms: (1) Elevated fumarate stabilizes HIF-1α/HIF-2α heterodimers via transcriptional and post-translational activation, driving tumorigenesis and metastasis; (2) competitively inhibits α-ketoglutarate-dependent histone demethylases (KDM), inducing epigenetic dysregulation; (3) promotes succinate-mediated DNA hypermethylation through TET enzyme inhibition (12). We identified a germline heterozygous *FH* variant (NM_000143.1: c.1240A>G; p. Lys414Glu) in exon 9, which led to the mutation of amino acid 414 of the coding protein from lysine to glutamate, first reported in 2024 (13). Although the identified FH missense variant (c.1240A>G, p.Lys414Glu) is currently classified as a variant of uncertain significance (VUS) in the ClinVar database, the patient’s overt clinical phenotype of FH-deficient tumors strongly supports its pathogenic role. This assertion is corroborated by previous reports: a case study described a patient carrying this variant with a strong family history of uterine fibroids (14), and a family with this variant presenting typical HLRCC features (skin and uterine fibroids, RCC) has also been documented. Furthermore, functional assays have demonstrated that the K414E mutation, when expressed in Fh1-null mouse cells, leads to a loss of fumarase activity and enhanced cell migration compared to FH wild-type (15). We recommend submitting this case data to the LOVD and ClinVar databases to facilitate the reclassification of this variant to likely pathogenic.

The incidence of HLRCC is low, with limited reported cases, and no international guidelines exist for diagnosis and treatment. FH protein immunohistochemistry (IHC) and germline genetic testing are key diagnostic tools for HLRCC. However, it is important to recognize that FH IHC alone may not universally reflect the underlying mutational status; certain missense variants can produce an aberrant protein with retained immunoreactivity, potentially leading to false-negative interpretations. In such contexts, 2-succinylcysteine (2SC) IHC—which detects fumarate-induced protein succination—is more widely accepted as a reliable functional surrogate for FH deficiency, irrespective of mutation type. Unfortunately, 2SC IHC could not be performed in our case because the corresponding tumor tissue blocks were unavailable. When it is found that patients with skin and uterine leiomyomas have kidney lesions, it is very important to have an annual renal magnetic resonance imaging test to detect early renal cell carcinoma (16). Because HLRCC-associated renal cell cancer (RCC) is highly invasive, annual monitoring of known HLRCC patients and removal of identified kidney neoplasms is recommended starting at age 10 (4).

The management of FH-deficient leiomyomas primarily involves surgical intervention and hormonal therapy (17). The first-in-human trial of the combination of bevacizumab and erlotinib in HLRCC patients was reported (18), demonstrating promising efficacy in this specific subtype. Emerging evidence supports the efficacy of VEGF/EGFR-targeted regimens (e.g., erlotinib/bevacizumab) in HLRCC-associated renal cell carcinoma (RCC) (19), with immunotherapy demonstrating significant improvements in overall survival (OS) for these patients (20). FH-deficient renal carcinoma exhibits compensatory metabolic defects due to disrupted TCA cycle and aerobic glycolysis dependence. FH-deficient HLRCC cells are sensitive to nicotinamide phosphoribose transferase (NAMPT) inhibition, a potential therapy for FH-deficient HLRCC-associated renal cell carcinoma (21). In addition, HLRCC-associated renal cell carcinomas are sensitive to mercaptopurine, a purine remediation pathway inhibitor (22). Current guidelines mandate germline *FH* mutation analysis, genetic counseling, and familial surveillance for HLRCC patients (23). While we recommended comprehensive family screening in this case, the patient declined genetic testing due to personal preferences. This disease usually shows invasive progression and has a poor prognosis, highlighting the urgent need for standardized treatment regimens in the management of advanced HLRCC-RCC (20).

Clinical evidence from our cohort demonstrates a persistent risk of distant metastasis in FH-deficient renal cell carcinoma (RCC) following curative-intent radical nephrectomy at early disease stages. Notably, FH-deficient/HLRCC-associated RCC currently lacks established therapeutic guidelines, with both immune checkpoint inhibitors and molecular-targeted agents requiring rigorous investigation through prospective clinical trials.

Several limitations of this case report should be acknowledged. First, the patient declined comprehensive family genetic screening, precluding a full assessment of familial segregation and hereditary risk among relatives. Second, due to exhaustion of available tumor tissue blocks and decalcification-related constraints, we were unable to perform 2-succinylcysteine (2SC) immunohistochemistry or sequence the tumor specimens for loss of the second FH allele; these analyses, if feasible, would have provided further confirmation of biallelic inactivation and functional FH deficiency. Finally, the follow-up period is relatively short given the aggressive nature of HLRCC-associated RCC, and long-term outcomes remain to be determined.

## 4 Conclusion

This case highlights the rare and aggressive nature of HLRCC-associated renal cell carcinoma, where early metastasis occurred despite radical surgery and systemic therapy. Immunohistochemical and genetic testing are essential for confirming FH deficiency. When young women present with multiple uterine leiomyomas and renal tumors, the possibility of HLRCC should be considered. Early genetic screening and multidisciplinary surveillance are critical for guiding treatment and improving prognosis.

## Data Availability

The original contributions presented in the study are included in the article/supplementary material. Further inquiries can be directed to the corresponding author.
